# A Review of Hypervolemic Hemodilution in Pediatric Neuroanesthesiology

**DOI:** 10.7759/cureus.89088

**Published:** 2025-07-30

**Authors:** Mohamad Taha, Rizq Imtiaz, Yumna Awwab, Sharif Mohamed

**Affiliations:** 1 Anesthesiology, Texas Agricultural and Mechanical (A&amp;M) University College of Medicine, Bryan, USA; 2 Biology, University of Houston-Clear Lake, Houston, USA; 3 General Medicine, California State Polytechnic University Pomona, Pomona, USA; 4 Anesthesiology, University of Texas Medical Branch, Galveston, USA

**Keywords:** blood conservation, cerebral autoregulation, craniosynostosis surgery, hypervolemic hemodilution, pediatric fluid management

## Abstract

Hypervolemic hemodilution (HVH) is a blood-sparing technique increasingly used in pediatric neuroanesthesiology, particularly during high-risk procedures such as craniosynostosis repair. By expanding intravascular volume with crystalloids or colloids before surgery, HVH dilutes red blood cell concentration, thereby reducing their loss due to bleeding, while maintaining cerebral oxygenation and hemodynamic stability. This approach is especially valuable in pediatric patients, who are more susceptible to transfusion-related complications due to their limited blood volume and immature immune systems. Compared to acute normovolemic hemodilution, HVH is simpler and equally effective when blood loss is anticipated to be moderate. However, its use requires careful monitoring, as HVH may affect intracranial pressure, cerebral autoregulation, and anesthetic pharmacokinetics. This review highlights the clinical applications, physiologic impacts, and safety considerations of HVH in pediatric neurosurgical care, while identifying gaps in current literature, such as the lack of long-term outcome data and high-quality comparative trials in pediatric patients.

## Introduction and background

In pediatric neurosurgery, particularly during procedures such as craniosynostosis repair or large tumor resections, intraoperative blood loss can be significant and quickly compromise tissue perfusion [1,2]. Unlike adults, children are more susceptible to subtle blood loss due to their smaller circulating volumes and limited physiologic reserve [1]. Despite the benefits of blood transfusions, there are potential risks, including both infectious and noninfectious complications, which occur more frequently in pediatric patients than in adults [3]. Common noninfectious risks include febrile non-hemolytic transfusion reactions, allergic reactions, transfusion-associated circulatory overload, transfusion-related acute lung injury, and both acute and delayed hemolytic reactions [4]. These noninfectious reactions can vary widely in severity, from mild, self-limited symptoms to severe, potentially life-threatening events. Blood transfusions are also associated with an increased risk of 30-day mortality [5].

While these events can affect patients of all ages, pediatric patients are particularly vulnerable due to their smaller blood volume and developing immune systems [6]. As a result, minimizing transfusion use in pediatric care is a growing focus in clinical settings [6]. A retrospective study of 133,671 transfusions at Vanderbilt University Medical Center found that pediatric patients experienced transfusion reactions at more than double the rate of adult patients (6.2 vs. 2.4 reactions per 1,000 transfusions) [7]. Allergic reactions, febrile non-hemolytic transfusion reactions, and hypotensive episodes were significantly more frequent in pediatric patients than in adults [7]. These findings suggest that children, due to their developmental physiology, are especially vulnerable to transfusion-related complications [7].

Hypervolemic hemodilution (HVH) is a relatively simple blood conservation technique that involves rapid intravascular volume expansion using 30-50 mL/kg of crystalloids (Ringer’s lactate) or colloids (albumin 5%) beyond maintenance requirements, estimated blood loss, and urine output [8]. It is a process that dilutes red blood cells by increasing plasma volume with cell-free fluids [8]. This results in a 20%-25% increase in circulating blood volume and a corresponding decrease in the concentration of cellular blood components [8]. This process replaces blood volume with cell-free fluid. It is considered “hemostatic” if blood volume is maintained within normal limits, or “acute” if done after induction of anesthesia and before surgical incision [9].

HVH can reduce the loss of red blood cells during surgical bleeding by diluting the blood before anticipated hemorrhage, resulting in increased tolerance to blood loss and a more stable balance between cerebral oxygen supply and demand throughout surgery [8,10]. Clinical outcomes have consistently demonstrated their efficacy and safety [8]. Currently, HVH is widely applied in various surgical fields, including thoracic, spinal, vascular, gastrointestinal, hepatic, urologic, and neurosurgical procedures such as craniosynostosis surgery [10]. Another advantage of this strategy is that it is easier to perform, requiring less equipment and fewer personnel, while effectively reducing the risk of blood contamination and maintaining the integrity of blood components [10].

In this review, we first outline the mechanism and physiological impact of HVH. We then compare it with acute normovolemic hemodilution (ANH), discuss pediatric-specific risks and considerations, and explore its application in craniosynostosis surgery. Finally, we address cerebral autoregulation (CA) and the role of intraoperative hemodynamic monitoring in ensuring safe and effective use of HVH during pediatric neurosurgical procedures. This review aims to highlight the clinical utility of HVH in pediatric anesthesiology and assess its potential as a safe, effective blood-sparing technique.

## Review

Methods

This article is a narrative review that aims to summarize current knowledge on HVH in pediatric neuroanesthesia. A structured literature search was conducted using PubMed. Search terms included combinations of “hypervolemic hemodilution”, “pediatric anesthesia”, “craniosynostosis”, “neurosurgery”, “blood conservation”, “transfusion”, and “cerebral autoregulation”. Reference lists from key articles were also manually reviewed for additional relevant sources. Studies were included if they addressed HVH or blood management strategies relevant to pediatric populations undergoing neurosurgery, or if they provided physiological or clinical rationale related to pediatric practice (including adult studies with relevant implications).

Mechanism of hypervolemic hemodilution

HVH is commonly used for perioperative blood conservation, as it allows for rapid intravascular volume expansion without significantly compromising hemodynamic stability [9,11]. Moreover, it enhances cerebral oxygen utilization, helps control surgical bleeding, and reduces the need for blood transfusion [9]. However, HVH can increase circulating blood volume, cerebral blood flow, and intracranial pressure, potentially leading to cerebral congestion and edema, disrupted cerebral oxygen metabolism, and decreased circulatory stability [9]. To reduce the effects of increased blood volume, anesthetics like sevoflurane are preferred because they cause vasodilation [12].

The use of HVH significantly alters the pharmacokinetics of propofol. In patients who underwent HVH, an increase in central compartment volume was observed, contributing to a lower plasma concentration of propofol in the first 10 minutes after administration [13]. This is partly due to hemodilution-induced reductions in plasma albumin levels, which increase the free, active fraction of propofol [13]. These changes affect the drug’s initial distribution and may influence anesthetic depth and onset [13,14]. Although propofol’s distribution half-life was not significantly shortened, the altered kinetics highlight the need for careful dose titration and monitoring during procedures involving HVH [13,14]. However, the study was limited by its small sample size (n=16) and its focus on adult patients undergoing hip replacement, which may limit its direct applicability to pediatric neurosurgical contexts [13].

Comparison with acute normovolemic hemodilution

ANH is a blood conservation technique proven effective in reducing the need for allogeneic blood transfusions [15,16]. When ANH is combined with erythropoietin, the capacity for allowable operative blood loss is increased [8]. Segal et al.’s meta-analysis showed moderate ANH efficacy across various surgical populations but included limited pediatric patients, highlighting a gap in pediatric neurosurgical data [16]. This technique involves collecting a predetermined volume of the patient’s blood immediately before surgery, storing it in an anticoagulant solution, and replacing the volume with colloid or crystalloid fluids to maintain hemodynamic stability [15,16]. Once major surgical bleeding has stopped, the autologous blood is reinfused, typically resulting in a higher final hematocrit at the end of the procedure [15]. Although ANH has been increasingly utilized in pediatric patients due to the decreased need for transfusions and the safety of using the patient’s blood, its use has been less extensively studied in this population [15,17].

Mathematical models comparing HVH, ANH, and no dilution have shown that for blood losses less than 40% of estimated blood volume (EBV), HVH and ANH result in nearly identical postoperative hematocrit levels (Table 1) [18]. Given its relative simplicity, HVH may be preferable over ANH in cases where blood loss is expected to remain below this threshold [18]. A randomized clinical trial involving 36 children undergoing scoliosis surgery compared HVH to standard management [6]. Patients in the HVH group received 12 mL/kg of Voluven [6]. While intraoperative blood loss was similar between the groups, those who underwent HVH required significantly less allogeneic blood transfusion (18 mL/kg vs. 28 mL/kg) [6].

**Table 1:** Key differences between hypervolemic and normovolemic hemodilution techniques HVH: hypervolemic hemodilution, ANH: acute normovolemic hemodilution

Technique	HVH	ANH
Mechanism	Intravascular volume expansion using crystalloids or colloids [8]	Preoperative blood removal with fluid replacement [15,16]
Blood storage needed	No [8]	Yes [15,16]
Hematocrit effect	Decreases to ~30% with volume loading [19]	Final hematocrit is typically higher after reinfusion [15,16]
Transfusion avoidance strategy	Dilutes blood prior to bleeding to reduce red cell loss [8]	Stores patient’s own blood for reinfusion post-bleeding [15,16]
Effectiveness	Similar postoperative hematocrit as ANH [18]	Similar postoperative hematocrit as HVH [18]
Clinical use	Simpler to perform, with less equipment and personnel required [10]	Less extensively studied in pediatrics [15,17]

Pediatric considerations and risks

Risk factors associated with poorer surgical outcomes include low body weight, large lesion size, extended operative duration, and perioperative anemia [5]. Excessive bleeding in these settings is often associated with hemodynamic instability, decreased cerebral perfusion and oxygenation, large-volume fluid administration, and a higher incidence of transfusion-related complications [5]. In stable pediatric ICU patients, adopting a restrictive transfusion strategy, typically by targeting a hemoglobin level of 7 g/dL rather than 9 g/dL, has been proven safe and is recommended as the standard practice [9].

There are also multiple limitations to the use of HVH in pediatric patients. Excessive hemodilution can significantly decrease the oxygen-carrying capacity of the blood, which may compromise oxygen delivery to vital organs [9]. This is a particular concern in infants and young children, whose metabolic demands are high relative to their circulating blood volume [1]. Additionally, HVH can lead to dilutional coagulopathy by reducing the concentration of clotting factors and platelets, which increases the risk of intraoperative and postoperative bleeding [20]. Pediatric patients may also be intolerant to hypervolemia itself, particularly those with cardiac or pulmonary comorbidities, increasing the risk of fluid overload and related complications such as pulmonary edema, impaired myocardial function, or prolonged mechanical ventilation [21,22]. Another major limitation of HVH is the risk of cerebral edema, which is of significant concern in children with impaired autoregulation or elevated baseline intracranial pressure [23,24]. In such patients, hypervolemia may exacerbate cerebral congestion and compromise perfusion [23,24]. Therefore, HVH should be applied cautiously or avoided entirely in pediatric patients with known intracranial hypertension, hydrocephalus, or cardiorespiratory comorbidities [23,24]. Establishing safe thresholds for hematocrit reduction and fluid volume administration is critical and should be guided by the patient's baseline hemoglobin, weight, neurologic status, and comorbid conditions [9].

When performing HVH, it is important to ensure that all intravenous fluids used for volume expansion are warmed to prevent hypothermia, a common perioperative complication that can worsen during surgery [25]. A careful, individualized evaluation of each patient beginning in the preoperative period is required to assess the risk of perioperative blood loss [9]. Hemoglobin and hematocrit levels must be reviewed and interpreted in the context of the patient’s age and weight [9]. Clinical signs of anemia or hypovolemia, as well as the presence of comorbidities, especially cardiac, pulmonary, or renal conditions, should be noted, as these may impact the patient’s ability to tolerate hemodilution or blood loss [9]. Moreover, the timing of HVH should be carefully planned in coordination with the surgical team, especially in cases involving cardiopulmonary bypass, to maintain hemodynamic stability and align with operative milestones [9]. Close intraoperative monitoring of volume status, coagulation parameters, and oxygenation is critical, as pediatric patients are less able to compensate for rapid shifts in intravascular volume [19]. Finally, the pediatric intensive care unit should be alerted in advance regarding the anticipated degree of postoperative HVH anemia to plan for postoperative monitoring, potential transfusion needs, and supportive care [9].

Intraoperative Fluid and Transfusion Management

Pediatric fluid management during blood salvage depends on the patient’s weight and blood loss [9]. For infants under 10 kg, rapid blood loss below 20% of EBV is managed with equal parts colloids and crystalloids [9]. When blood loss exceeds 20%, red blood cell transfusions are necessary to maintain oxygen delivery and hemodynamic stability while avoiding fluid overload and excessive dilution [9]. There is no clear evidence favoring colloids over crystalloids initially, but colloids may carry increased risks if the blood-brain barrier is impaired [9].

Decisions regarding blood transfusion take into consideration hematocrit, clinical condition, patient weight, and bleeding severity [9]. For example, infants with ongoing bleeding and hematocrit between 21% and 25% typically require transfusion, whereas older stable children may not [9]. Transfusion volume is calculated based on weight and desired hemoglobin increase [9]. Platelet transfusions are indicated when counts fall below 50,000-100,000/μL, with standard doses raising levels by 20,000-50,000/μL per unit [9]. Fresh frozen plasma can be used to correct clotting abnormalities [9]. Moreover, antifibrinolytic agents, especially tranexamic acid (TXA), are used to help reduce bleeding and transfusion needs [9,26]. TXA is typically given prophylactically for expected blood loss over 40% EBV, with a loading dose of 10 mg/kg over 15 minutes followed by a maintenance infusion of 5 mg/kg per hour (Table 2) [9].

**Table 2:** Pediatric thresholds and dosing parameters relevant to blood conservation HVH: hypervolemic hemodilution, TXA: tranexamic acid

Parameter	Value
Target hematocrit during craniosynostosis	~30% [19]
Hematocrit for considering transfusion (infants with ongoing bleeding)	21–25% [9]
HVH target hematocrit	~25–30% [19]
HVH fluid dose (colloid)	~15–20 mL/kg [9]
TXA loading dose	10 mg/kg over 15 minutes [9]
TXA maintenance dose	5 mg/kg/hr [9]
Platelet transfusion trigger	50,000–100,000/μL [9]
Platelet count increases per unit	20,000–50,000/μL [9]
Fluid choice for blood loss <20% EBV (infants <10 kg)	Equal parts crystalloids and colloids [9]

Use in craniosynostosis surgery

Craniosynostosis is a condition affecting approximately one in 2000-2500 live births in which one or more cranial sutures fuse prematurely, most often spontaneously, but occasionally due to syndromic or familial causes [27]. Its repair is a pediatric neurosurgical procedure frequently complicated by significant and rapid blood loss, resulting in major anesthetic challenges [19,28]. In infants with limited blood volume, the potential for serious hemorrhage requires careful perioperative management, including constant monitoring and timely blood transfusions [19,28]. Accurate estimation of blood loss is particularly difficult during these surgeries, as factors such as irrigation fluids and cerebrospinal fluid can affect measurements such as sponge weights and suction volumes [19]. Surgical blood loss may reach 20-500% of total blood volume in a short period of time, highlighting the need for accurate fluid and transfusion management [29,30]. Several operative and anesthetic factors influence bleeding, including the choice of ventilation method, use of induced hypotension, and local vasoconstrictive agents [19]. Surgical duration and the number of cranial sutures involved also impact hemorrhage severity, with bicoronal procedures typically associated with greater blood loss than unicoronal or sagittal repairs [19].

Given these challenges, maintaining adequate oxygen delivery while minimizing blood transfusion is essential in pediatric patients undergoing craniosynostosis surgery [19]. HVH as a blood conservation strategy is particularly relevant here. By expanding intravascular volume with crystalloids or colloids before blood loss occurs, HVH lowers hematocrit and reduces red cell loss during hemorrhage [19]. This approach supports hemodynamic stability and cerebral oxygenation, which is important in infants with limited physiologic reserves [19]. Furthermore, strategies such as continuous communication with surgeons, active monitoring of vital signs, and transfusion protocols aiming for a target hematocrit around 30% can help optimize patient outcomes [19]. Coombs et al. conducted a systematic review of 52 studies, highlighting the variability in blood management protocols and surgical outcomes; however, the analysis was limited by the lack of direct prospective comparisons between HVH and other techniques [30]. Incorporating HVH into anesthetic management for craniosynostosis procedures could therefore enhance blood conservation and reduce transfusion-related risks in this vulnerable population [19]. However, due to the complexity of blood loss and cerebral physiology in these surgeries, individualized evaluation and careful intraoperative monitoring remain important [19,28].

Cerebral autoregulation

CA is highly variable in children due to age-dependent differences in cerebral blood flow, arterial blood pressure, and brain metabolism [23,24]. This variability complicates intraoperative management during pediatric neurosurgery, especially when techniques like HVH are used to conserve blood [23]. When CA is impaired or disrupted, such as during significant volume shifts or hemodynamic instability, cerebral perfusion may become pressure-passive [23]. In this state, blood flow directly follows changes in the arterial blood pressure, which increases the risk of cerebral congestion, ischemia, or secondary brain injury [23]. This is a significant concern when using HVH, as this technique increases intravascular volume and potentially intracranial pressure [9].

Hemodynamic Monitoring

A recent study evaluating the hemodynamic impact of acute HVH using a 15 mL/kg fluid bolus found that this volume consistently increased preload and cardiac output while reducing afterload, without altering cardiac contractility [31]. These changes were assessed using a transesophageal Doppler (TED) device, which provided real-time monitoring of parameters such as stroke volume, cardiac index, and systemic vascular resistance, which are essential to monitor in the setting of hemodilution [31,32]. TED also carries fewer risks and complications compared to more invasive devices like pulmonary artery catheters, allowing for safer and continuous assessment during surgery [32,33]. The most sensitive indicators of volume change were stroke index and central venous pressure, which responded early during fluid administration [31]. While some prior studies used larger volumes over longer durations, this study confirmed that a standard HVH volume of 15 mL/kg infused over 10 minutes is hemodynamically safe and well-tolerated under general anesthesia [31]. Notably, despite a reduction in hematocrit, arterial oxygen content remained within normal limits, suggesting preserved oxygen delivery [31]. The data also highlighted that while contractility indices remained stable, afterload reduction, likely due to decreased blood viscosity, played a key role in increasing cardiac output [31]. These findings support the use of TED as a valuable tool for real-time intraoperative monitoring during HVH and demonstrate the safety of modest-volume HVH, although the small sample size and focus on an adult surgical population may somewhat limit the applicability of these results to pediatric practice [31].

Additional Monitoring Tools

Adjunct cerebral monitoring tools such as near-infrared spectroscopy (NIRS) and jugular bulb oximetry can also be useful in this setting. NIRS provides non-invasive, indirect measurements of regional cerebral oxygen saturation, helping identify early signs of hypoperfusion or over-resuscitation [34]. Jugular bulb oximetry, though more invasive, measures the global metabolic demand of the brain compared to its oxygen supply and can help guide transfusion decisions by detecting imbalances between oxygen needs and delivery during hemodilution [35]. Furthermore, intraoperative monitoring of volume and coagulation status is also critical in pediatric neurosurgery due to the narrow margin for physiologic compensation. Volume status is typically assessed using invasive arterial lines for continuous blood pressure monitoring and frequent labs, while conventional labs (PT, aPTT, fibrinogen, platelets) are routinely used for coagulation [36,37]. Viscoelastic tests such as thromboelastography or thromboelastometry are also available and can offer a more dynamic assessment [37].

Limitations

Due to the limited number of studies and the heterogeneity of available data, no formal statistical analyses, such as meta-analyses or meta-regression, were performed. Consequently, quantitative assessments of effect size, heterogeneity, and publication bias are not included. Because pediatric neuroanesthesia-specific data are limited, some findings were drawn from adult or non-neurosurgical pediatric studies. Therefore, further comparative studies and systematic reviews are needed to better establish the safety and efficacy of HVH in this population.

## Conclusions

HVH represents a safe and effective strategy for perioperative blood conservation in pediatric neurosurgery. By expanding intravascular volume and reducing hematocrit before anticipated blood loss, HVH improves cerebral oxygen delivery, reduces reliance on blood transfusions, and supports hemodynamic stability, which are critical considerations in children with limited physiologic reserve. Clinically, HVH is most appropriate for surgeries with high blood loss potential, such as craniosynostosis repair. Continuous invasive hemodynamic and coagulation monitoring should guide fluid administration and transfusion decisions throughout the procedure. Early communication with the surgical and pediatric intensive care teams supports coordinated perioperative management.

Despite its broad range of use in surgery and favorable safety profile, HVH requires careful application in pediatric patients, especially in cases with large-volume shifts and procedures with elevated risk for cerebral congestion or impaired autoregulation. CA in children is variable and difficult to monitor; thus, individualized risk assessment and careful fluid management are essential. The use of real-time cerebral hemodynamic monitoring and CA-guided strategies is one method of improving the safety of HVH in this patient population. Future research should focus on evaluating the long-term outcomes of HVH-based protocols in neurosurgery.
